# Molecular screening of transitional B cells as a prognostic marker of improved graft outcome and reduced rejection risk in kidney transplant

**DOI:** 10.3389/fimmu.2024.1433832

**Published:** 2024-08-12

**Authors:** Inés Perezpayá, Sergio G. Garcia, Marta Clos-Sansalvador, Marta Sanroque-Muñoz, Miriam Font-Morón, Paula Rodríguez-Martínez, Anna Vila-Santandreu, Jordi Bover, Francesc E. Borràs, Laura Cañas, Marcella Franquesa

**Affiliations:** ^1^ REMAR-IGTP Group, Germans Trias i Pujol Research Institute (IGTP) & Nephrology Department, University Hospital Germans Trias i Pujol (HUGTiP), Barcelona, Catalonia, Spain; ^2^ Department of Cell Biology, Physiology and Immunology, Autonomous University of Barcelona, Bellaterra, Spain; ^3^ Department of Biochemistry and Molecular Biology, Autonomous University of Barcelona, Bellaterra, Spain; ^4^ Pathology Department, University Hospital Germans Trias i Pujol (HUGTiP), Barcelona, Catalonia, Spain; ^5^ Department of Cell Biology, Physiology, and Immunology, Universitat de Barcelona (UB), Barcelona, Spain

**Keywords:** acute graft rejection, flow cytometry, immunophenotyping, transitional B cells, Breg

## Abstract

**Introduction:**

Understanding immune cell dynamics in kidney transplantation may provide insight into the mechanisms of rejection and improve patient management. B cells have gained interest with a special relevance of the “regulatory” subsets and their graft outcome prognostic value. In this study, we aimed to prove that the direct immunophenotyping and target gene expression analysis of kidney transplant patients' fresh whole blood will help to identify graft rejection risk and assist in the monitoring of kidney transplanted patients.

**Methods:**

We employed flow cytometry and qPCR techniques to characterize B and T cell subsets within fresh whole blood samples, with particular emphasis on transitional B cells (TrB) identified as CD19^+^CD24^hi^CD38^hi^. TrB are a relevant population in the context of kidney transplantation and are closely associated with regulatory B cells (Bregs) in humans. Patients were monitored, tracking pertinent clinical parameters and kidney-related events, including alterations in graft function and episodes of biopsy proven rejection.

**Results:**

Higher percentages of TrB cells at 3 months after transplantation were positively associated with better graft outcomes and lower biopsy-proven acute rejection risk. Furthermore, a novel panel of B cell regulatory associated genes was validated at 3 months post-transplantation by qPCR analysis of peripheral blood mononuclear cell (PBMC) mRNA, showing high predictive power of graft events and prognostic value.

**Discussion:**

These findings suggest that monitoring TrB may provide interesting patient management information, improve transplant outcomes, and allow for personalized drug regimens to minimize clinical complications.

## 1 Introduction

B cells’ canonical role in humoral immunity is through antibody-mediated responses. However, they have other important non-antibody-mediated effector functions such as antigen presentation, T-cell activation, cellular co-stimulation, and cytokine secretion of tumor necrosis factor (TNF)-α, interferon gamma, or interleukin (IL)-6 (1–3).

B cells have a regulatory role in immune response modulation through diverse mechanisms. This regulatory function is mainly attributed to a B-cell subset called regulatory B cells (Bregs). Bregs primarily regulate the immune response by secreting cytokines, particularly IL-10, transforming growth factor (TGF)-β, IL-35, and Granzyme B, which suppress pro-inflammatory lymphocyte differentiation, reduce TNF-α production, inhibit lymphocyte proliferation, and promote the expansion of FoxP3+ regulatory T cells (Tregs) (1–4).

Despite the interest in their regulatory role, a unique phenotype or transcriptional marker has not yet been defined for Bregs. They constitute a heterogeneous cell population with a regulatory potential that can be identified at different stages of B-cell development (1, 3, 5). Different phenotypes have been described with two main signatures defined in humans: CD19^+^CD24^hi^CD38^hi^ or transitional B-cell phenotype (TrB) (6–8) and CD19^+^CD5^+^CD1d^hi^ (9). Other interesting surface markers such as CD9 and T-cell immunoglobulin mucin receptor (TIM)-1 have also been recently described (10, 11).

In the context of kidney transplantation, Bregs have been positively linked to allograft tolerance, being higher in operationally tolerant transplant recipients compared to patients with chronic rejection or stable graft function on immunosuppressive treatment (12, 13). On the other hand, the depletion of Bregs with Rituximab, especially in the peri-transplant period, has been shown to increase the risk of acute cellular rejection, suggesting that Bregs depletion might enhance the alloimmune response by losing their regulatory role and favoring an inflammatory state (14, 15). Furthermore, several studies have demonstrated that patients with lower Bregs during follow-up had worse graft outcomes and higher risk of rejection (16–19), suggesting its utility as a biomarker of clinical outcome and risk stratification.

In a previous work, we identified a panel of human genes differentially overexpressed *in vitro* by Bregs induced by co-culture with mesenchymal stromal cells (20). B cells cocultured with mesenchymal stromal cells had enrichment of TGF-β-associated pathways, and blockage of TGF-β signaling abrogated both TrB cell phenotype expression and the induction of regulatory B cells, and inhibited regulatory B-cell function. Based on the relation between this pathway and the regulatory functions of B cells *in vitro*, we hypothesized that the TrB-associated signature could represent a novel approach to the monitoring of Breg.

In this work, we hypothesize that the characterization and quantification of fresh whole blood transitional B cells by flow cytometry (CD19^+^CD24^hi^CD38^hi^) constitutes a valuable tool to stratify kidney transplant patients according to their risk of rejection or allograft outcome, being higher in patients with better graft outcome and no rejection during the first year after transplantation. In addition, we ought to validate a novel panel of Breg *in vitro*-associated genes to test its prognostic potential.

## 2 Materials and methods

### 2.1 Patients

**Figure 1:**
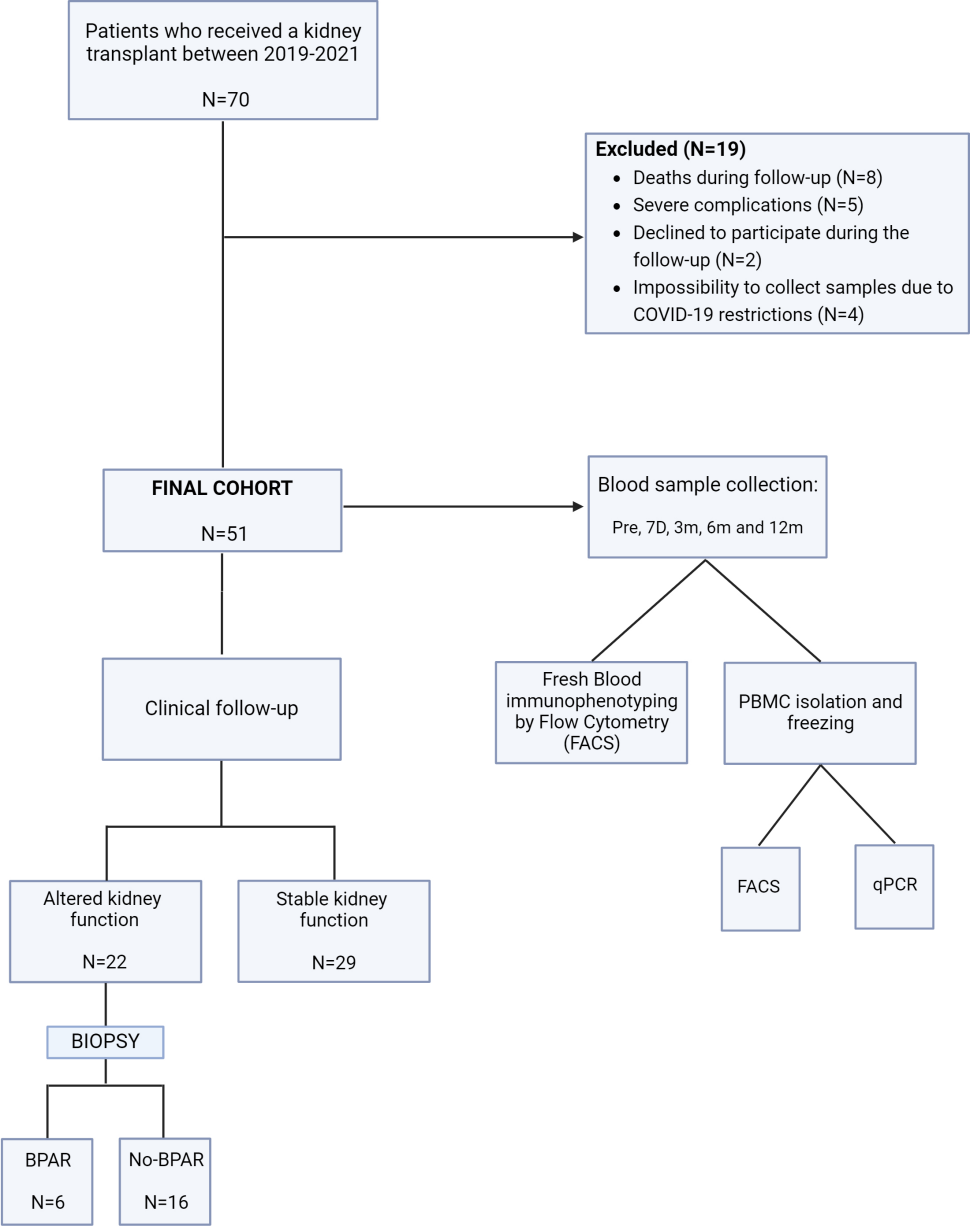
Schematic showing the study design, participants, and methods. Patients from the Germans Trias I Pujol hospital were enrolled in this study. BPAR, biopsy-proven acute rejection. no-BPAR, patients with altered kidney function but no diagnosis of acute rejection in biopsies; qPCR, quantitative polymerase chain reaction; Pre, prior to transplantation; 7D, seven days; 3/6/12m, 3/6/12 months.

A total of 70 unselected recipients of kidney transplantation were included in this prospective, single-center, incident cohort study between May 2019 and July 2021. Sample size was calculated using Wilcoxon–Mann–Whitney test assuming an *α*-error of 0.05 and a power of 0.8 with an effect size of 1.13, obtaining a minimum sample size of 14 per group (G*Power software, Düsseldorf University). Patients from the Germans Trias i Pujol Hospital were followed up over 12 months post-transplantation. During follow-up, 19 patients were excluded from the study; thereby, 51 patients formed the final cohort. Specifically, eight patients passed away, while five were excluded due to early graft loss resulting from surgical complications requiring transplantectomy (three patients) or non-treatable acute rejection within the first 3 months due to the severity of their clinical condition (two patients). Additionally, four patients who were initially included were excluded due to the impossibility to collect samples during follow-up due to COVID-19 restrictions. Two patients declined to participate in the study during follow-up. None of the patients mentioned were included in the subsequent analysis, as illustrated in the workflow outlined in **Figure 1**. The study was performed according to the Declaration of Helsinki, and local ethics committee approval was granted (PI-19-019). Informed consent was obtained from all participants.

### 2.2 Demographic data and protocol

The inclusion criteria for the enrollment of patients included being older than 18 years old and receiving an ABO-compatible graft. The demographic features of the cohort are summarized in **Table 1**. Each patient received immunosuppressive therapy according to our center protocol. Induction therapy with rATG (Thymoglobulin^®^; Genzyme Corporation, Cambridge, MA) was given within the first week at an intended cumulative dose of 6 mg/kg to patients with high immunological risk, defined as patients receiving a second kidney transplant or recipients with historic complement-dependent cytotoxicity (CDC)-based panel reactive antibodies (PRAs) >30%. All other patients, defined as low-risk patients, received 20 mg of basiliximab (Simulect^®^; Novartis, Basel, Switzerland) on days 0 and 4. Low-risk patients receiving kidney transplant from HLA-haploidentical living-related donors did not receive any induction therapy (*n* = 2).

**Table 1:** Baseline demographic and clinical characteristics of the study cohort and baseline characteristics of patients that had a kidney biopsy done during follow-up, classified based on histology phenotype in biopsy-proven acute rejection (BPAR) and no biopsy-proven acute rejection (No-BPAR).

	Total (*n* = 51)	Stable kidney function (SKF) (*n* = 29)	Altered kidney function (AKF) (*n* = 22)	*p*-value	BPAR (*n* = 6)	NoBPAR (*n* = 16)	*p*-value
**Age**	60 (35–80)	61 (36–80)	58 (35–74)	0.36	59 (55–63)	58 (35–74)	0.47
**Sex (male)**	24 (47%)	16 (55%)	11 (40.7%)	0.13	2 (33%)	9 (56.3%)	0.40
**IS treatment Pre-KTx**	10 (19.6%)	6 (20.7%)	4 (18.2%)	0.71	0	4 (25%)	0.37
**Previous KTx**	6 (11.8%)	3 (10.3%)	3 (13.6%)	0.71	0	3 (18.8%)	0.23
**CKD etiology** Unknown PKD Glomerulonephritis^a^ DN Others^b^	20 (39.2%) 13 (35.5%) 7 (13.7%) 4 (7.8%) 7 (13.7%)	14 (48%) 7 (24%) 2 (6.8%) 1 (3%) 5 (17%)	6 (27%) 6 (27%) 5 (22.5%) 3 (13%) 2 (9%)	0.37	1(16.7%) 3 (50%) 0 1 (16.7%) 1 (16.7%)	5 (31.3%) 3 (18.8%) 3 (18.8%) 2 (12.5%) 3 (18.8%)	0.48
**Dialysis**	35 (68.6%)	21 (72.4%)	14 (63.6%)	0.5	4 (66.7)	10 (62.5)	0.77
**Months in dialysis**	8 (0–280)	22 (0–240)	12.6 (0–72)	0.27	2 (0–72)	9.6 (0–64)	0.73
**Type of donor** After brain death After circulatory death Live donor	24 (47%) 20 (39%) 7 (13.7%)	13 (44.8%) 12 (41%) 4 (13.7%)	11 (50%) 8 (36.4%) 3 (13%)	0.81	2 (33%) 3 (50%) 1 (16.7%)	8 (50%) 5 (31.3%) 3 (18.8%)	0.72
**CIT (min)**	860 (81–1,605)	832 (81–1,605)	735 (140–1,500)	0.64	720 (210–1,125)	752 (140–1,500)	0.76
**HLA mismatches** 0–3 4–6	10 (19.6%) 41 (80.4%)	4 (13.8%) 25 (86%)	6 (27.3%) 16 (72.7%)	0.23	2 (33%) 4 (66.7%)	4 (25%) 12 (75%)	0.76
**Anti-HLA Abs pre-KTx**	26 (51%)	14 (48%)	12 (54%)	0.65	6 (100%)	6 (37.5%)	0.001**
**DSA pre-KTx**	3 (6%)	1 (3.4%)	2 (9.1%)	0.39	2 (33.3%)	0	0.01*
**DSA *de novo***	3 (6%)	0	3 (13%)	0.04*	1 (16.7%)	2 (12.5%)	0.06
**Anti-HLA Abs *de novo***	4 (7.8%)	1 (3.4%)	3 (13%)	0.24	1 (16.7%)	2 (12.5%)	0.61
**Induction therapy** rATG Basiliximab None	25 (49%) 24 (47%) 2 (3.9%)	12 (41%) 17 (58%) 0	13 (59%) 7 (31.8%) 2 (9%)	0.06	4 (66.7) 1 (16.7) 1 (16.7)	9 (56.3) 6 (37.5) 1 (6.3)	0.52
**Maintenance therapy** PDN + FK + MMF PDN + FK + i-mTOR	46 (90.2%) 5 (9.8%)	25 (86%) 4 (13.8%)	21 (95.5%) 1 (4.5%)	0.27	6 (100%) 0	15 (93.8%) 1 (6.3%)	0.63
**Delayed graft function^1^**	11 (21.6%)	6 (20.7%)	5 (22.7%)	0.86	1 (16.7%)	4 (25%)	0.62
**CMV infection**	10 (19.6%)	4 (13.8%)	6 (27.3%)	0.23	1 (16.7%)	5 (31.3%)	0.44
**SARS-CoV-2 infection**	6 (11.8%)	3 (10.3%)	3 (13.6%)	0.71	2 (33%)	1 (6.3%)	0.11
**Bacterial infection** **first week post-transplant**	15 (29.4%)	4 (37.5%)	8 (37.5%)	0.09	3 (50%)	5 (31.25%)	0.32
**Creatinine (mg/dL)** 1 week 3m 6m 12m	2 (0.6–12) 1.40 (0.7–8.7) 1.50 (0.7–3.4) 1.44 (0.6–3.2)	2.8 (0.74–12) 1.33 (0.71–2.4) 1.39 (0.7–2.7) 1.35 (0.6–2.7)	3.4 (0.6–12) 2.05 (0.9–8.7) 1.81 (0.9–3.5) 1.79 (1–3.2)	0.17 0.02* 0.01* 0.005**	2.7 (1–12) 1.62 (1–2.6) 1.6 (1–3.47) 2.07 (1.1–3.1)	2.2 (0.68 9.16) 1.79 (0.8–8.7) 1.59 (0.8–3.2) 1.64 (1–2.9)	0.73 0.79 0.79 0.5
**Proteinuria (mg/g)** 3m 6m 12m	235 (21–3,085) 173 (15–3,521) 177 (13–2,972)	225 (21–710) 192 (15–928) 210 (13–682)	710 (108–3,085) 582 (75–3,521) 424 (95–2,972)	0.07 0.015* 0.3	292 (108–1,227) 436 (98–949) 183 (98–459)	316 (145–3,085) 210 (75–3,521) 177 (95–2,972)	0.59 0.79 0.54

^1^Dialysis required within the first postoperative week. ^a^IgA, membranous and focal segmental. ^b^Lupus, vasculitis and hypertensive nephrosclerosis. IS, immunosuppressive; KTx, Kidney transplant; BPAR, biopsy-proven acute rejection; CKD, chronic kidney disease; PKD, polycystic kidney disease; DN, diabetic nephropathy; CIT, cold ischemia time; Abs, antibodies; HLA, human leukocyte antigen; DSA, donor-specific antibodies; PDN, prednisolone; FK, tacrolimus; MMF, mycophenolate mofetil; imTOR, everolimus; CMV, cytomegalovirus. Values are median (range) for continuous variables or number (%) for categorical variables. p-value significant < 0.05 (bilateral). All categorical variables were compared using the chi-square test and Fisher’s exact testing. Continuous variables are analyzed using Student’s t-test if comparing two groups or ANOVA with Tukey post hoc test for multiple comparisons if normally distributed and the Mann–Whitney test for variables with skewed distribution. p < 0.05 are indicated as bold values.

The main maintenance therapy received was corticoids (PDN) (methylprednisolone Day 1: 500 mg, Day 2: 125 mg; from Day 3, 1 mg/kg per day of PDN tapered to 5 mg by month 2 post-transplantation), mycofenolate mofetil (MMF) (Myfortic^®^ 720 mg twice/day reducing to 360 mg twice/day by 2 months post-transplantation), and tacrolimus (FK) (predose levels of 7–10 ng/mL during the first 3 months, then 5–8 ng/mL). Patients with high oncological risk or past history of BK polyomavirus or cytomegalovirus (CMV) infection and with no prior diagnosis of focal segmental glomerulosclerosis as cause of ESRD received mTOR inhibitor, everolimus (target trough levels of 3–6 ng/mL), prednisolone, and tacrolimus as maintenance therapy (trough levels of 4–6 ng/mL).

Patients with altered kidney function (AKF) were identified during follow-up according to the following criteria: increase in serum creatinine of 25% above the baseline confirmed in at least two determinations excluding other clinical explanations (21) and/or increased proteinuria ≥0.5 g/day confirmed in at least two determinations assessed by urine protein–creatinine ratio, failure to decrease 10% serum creatinine levels on three consecutive days, and/or requirement of dialysis after the second post-operative week with no other clinical explanation (22). Since it was not established in our center to perform biopsies defined by protocol, for-cause biopsies were performed only to patients with AKF. Other complications were assessed, such as acquiring an active CMV or SARS-CoV-2 infection during follow-up. Based on biopsy diagnosis, patients with signs of acute rejection were classified as biopsy-proven acute rejection (BPAR), while those without acute rejection were classified as noBPAR.

For long-term study, the cohort of patients was followed-up for an average period of 45.5 months (range: Patient #1: 59 months to Patient #51: 33 months) during which clinical follow-up and events, biopsies, and pathological diagnoses were recorded.

### 2.3 Whole blood processing

Peripheral blood was collected in 10-mL EDTA test tubes before transplantation and at 1 week, 3 months, 6 months, and 12 months after transplantation. Whole blood (200 μL) was used for direct immunophenotyping, and the rest was processed to isolate peripheral blood mononuclear cells (PBMCs) by density gradient separation using Ficoll-Paque (GE Healthcare, Uppsala, Sweden). PBMCs were counted by using a hemocytometer and Trypan blue staining and frozen in a solution of 90% heat-inactivated fetal bovine serum (Lonza) and 10% dimethyl sulfoxide (DMSO) at a maximum of 10 million cells per vial and stored at −190°C for further use.

### 2.4 Fresh whole blood cellular immunophenotyping

**Figure 2:**
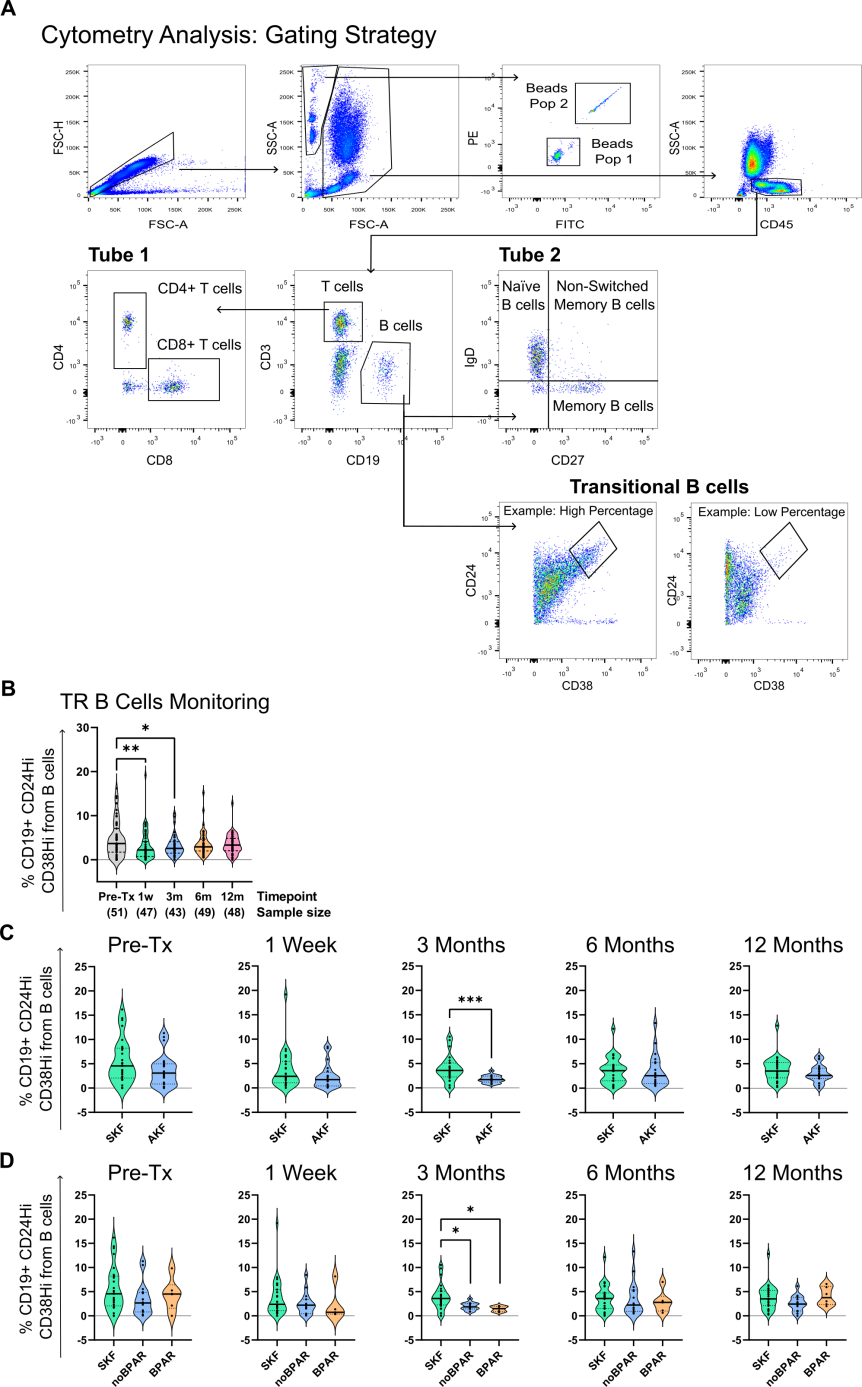
Transitional B-cell (CD19^+^CD24^hi^CD38^hi^) percentages are reduced at 3 months post-transplantation in kidney transplanted patients with altered kidney function (AKF) while remain stable in patients with stable kidney function (SKF). **(A)** Cytometry gating strategy. First, singlets are gated by forward scatter (FSC-H vs. FSC-A). From singlets, alive cells and beads are gated separately. Beads are further divided into the two different populations of beads that compose the mix. Alive cells are further gated by CD45 expression. From CD45+, T (CD3^+^CD19^-^) and B cells (CD3^-^CD19^+^) are gated. T-cell CD4 and CD8 populations were analyzed in tube 1. In tube 2, from total B cells, we analyzed transitional B-cell populations (CD19^+^CD24^hi^CD38^hi^), memory (CD19^+^CD27^+^IgD^-^), and naïve B cells (CD19^+^CD27^-^IgD^-^). **(B)** Representation of the percentage of transitional B cells (TrB) from total B cells at all time points of study. Transitional B-cell percentages are reduced at 1 and 3 months. **(C)** Comparison of transitional B-cell percentages between SKF and AKF groups. **(D)** Comparison of transitional B-cell percentages between patients with SKF and AKF with diagnosis of biopsy-proven acute rejection (BPAR) or those who do not (noBPAR). There was a significant reduction at 3 months in patients from the SKF group compared to AKF. * *p* < 0.05, ** *p* < 0.01, *** *p* < 0.001.

Immunophenotyping was performed in fresh whole blood by flow cytometry. Samples were labeled with antibodies in two independent polypropylene test tubes (antibody panel, commercial manufacturer, and clones can be found in **Supplementary Table 1**). Tube 1 was used to analyze B- and T-cell populations and perform absolute cell counts (number of cells/µl whole blood) by the addition of Perfect-Count Microspheres™ (Cytognos) without washing steps. We analyzed B-cell subpopulations in tube 2 and extrapolated the results from absolute B cells in tube 1 to obtain absolute counts from B-cell subsets (**Figure 2A**). Frequencies (% from a higher subset) were also assessed.

Whole blood (50 or 100 µL) was added by reverse pipetting to tubes 1 and 2, respectively. Negative control with no labeling was performed for each sample. Samples were incubated with antibodies for 20 min at room temperature. Red blood cells were lysed with FACS Lysing 1× (BD Pharma LyseTM). Samples were analyzed using a flow cytometer FACS CANTO II (BD).

### 2.5 Frozen PBMC immunophenotyping and RNA extraction

PBMCs were thawed and resuspended in 1 mL of PBS 1×. After cell counting by using a hemocytometer and trypan blue staining, 200 µL of cell solution was added to tube 1 and tube 2, and 100 µL was added to blank from the same antibody panel and protocol from the “Fresh whole blood cellular immunophenotyping” section, without Perfect-Count Microspheres.

RNA was extracted from the remaining 500 µL using the RNeasy Mini kit (Qiagen) following the manufacturer’s protocol with no β-mercaptoethanol or DNase treatment. RNA quality and concentration were assessed by nanodrop and Agilent 2200 Bioanalyzer System with a High Sensitivity RNA ScreenTape^®^ analysis.

### 2.6 Gene expression analysis by qPCR

For qPCR analysis, whole RNA content was used for cDNA synthesis by using random hexamers (Qiagen) and the One-step RT-PCR kit (Bio-Rad Laboratories) according to the manufacturer’s instructions using the Applied Biosystems 2720 thermal cycler. qPCR was performed using the “Master Mix PowerUp SYBR green” kit (Thermo Fisher Scientific) and analyzed on the LightCycler 480 II-Real-Time PCR system (Roche). Primer sequences can be found in **Supplementary Table 2**. Samples were analyzed in triplicate; 1 ng of cDNA was used for each triplicate. GAPDH results were used as endogenous reference values to quantify the relative expression of each marker by the 2^−ΔΔCT^ method relative to the AKF or the noBPAR groups.

### 2.7 Statistical analysis

Statistical analysis was performed using GraphPad Prism v9 (GraphPad Software La Jolla, CA, USA) and IBM SPSS statistics (IBM, Ehningen, Germany).

Data are presented as percentages (percentage of cell population from parent cell gate) or cell counts (number of cells/µL of whole blood). Data normality was tested using the Shapiro–Wilk, Kolmogorov–Smirnov, D’Agostino–Pearson, and Anderson–Darling tests. No outliers were discarded from the analysis. For the analysis of clinical variables, all categorical variables were compared using the chi-square test and Fisher’s exact testing, and continuous variables were analyzed using Student’s *t*-test or Mann–Whitney test. Analyses with multiple comparisons were completed using one-way ANOVA with Tukey *post-hoc* analysis in the case of normally distributed variables or the Kruskal–Wallis test with Dunn’s multiple comparison tests for variables with a skewed distribution.

The percentage of missing data for each time point was 0%, 5.88%, 15.69%, 3.92%, and 3.92% for time points pre-transplantation, 1 week, 3 months, 6 months, and 12 months post-transplantation, respectively. Receiver operating characteristic (ROC) curves and area under the curve (AUC) calculations were used to evaluate the potential of TrB cells to discriminate patients between groups by the Wilson/Brown method. Survival curves were compared by the Log-rank (Mantel-Cox) test. Spearman correlation coefficient was used to assess linear correlations. Combined ROC curve AUC values were obtained by multiple logistic regression, and VIF values were checked to discard multicollinearity and were <2 for all variables. *p*-values ≤0.05 were considered significant for all tests. Data analysis was performed using GraphPad Prism v10 and SPSS (version 25) and figures were created using Inkscape v.1.2.

## 3 Results

### 3.1 Higher % of CD19^+^CD24^hi^CD38^hi^ transitional B cells at 3 months correlate with improved graft outcome

First, we studied the evolution of transitional B cells (TrB cells) after transplantation by flow cytometry (**Figure 2A**) and we found that at 1 week and 3 months, TrB cell percentage from total B cells was significantly lower than at pre-transplantation (4.717% ± 4.115% vs. 3.152% ± 3.415% vs. 1 week and 3.089% ± 2.344% vs. 3 months) (**Figure 2B**).

To analyze the association between CD19^+^CD24^hi^CD38^hi^ TrB cells and graft outcome, we classified patients into two groups: patients with stable kidney function (SKF) and patients with an AKF during follow-up (see **Table 1** for clinical data and Materials and Methods for criteria). There was a statistically significant difference in the percentage of CD19^+^CD24^hi^CD38^hi^ TrB cells being higher at 3 months (**Figure 2B**) in patients with SKF compared to patients with AKF (*p* = 0.0012, Mean % TrB cells of 3.845% ± 2.627% vs. 1.811% ± 0.7794%). No statistical significance was found at other time points (**Figure 2C**).

The absolute number of TrB cells at 3 months was also significantly different between the SKF and AKF groups (*p* = 0.0423, Mean TrB cells/µL of total blood of 3.536 ± 2.663 vs. 1.948 ± 1.214). No statistical significance was found at other time points (data not shown).

Correlation to graft outcome during follow-up was also seen in common clinical parameters. In all time points, creatinine and proteinuria were higher in patients with AKF, except for proteinuria at 12 months (**Table 1**).

To further analyze TrB cell potential to predict graft outcome beyond graft function, we stratified patients with AKF, based on the histological findings in patients who had a BPAR (See **Supplementary Table 3** for all kidney biopsy histological diagnosis) and those with no evidence of rejection (noBPAR) (see Materials and Methods and **Table 1**). TrB cell percentage at 3 months was significantly lower in both the noBPAR (*p* = 0.0438, Mean% TrB cells of 3.845% ± 2.627% vs. 1.982% ± 0.8242%) and BPAR groups compared to SKF (*p* = 0.0286, Mean% TrB cells of 3.845% ± 2.627% vs. 1.434% ± 0.5672%) (**Figure 2D**). The BPAR group had the lowest TrB cell total numbers at 3 months of all three groups, but no significant difference was found between noBPAR and BPAR groups (*p* = 0.99, Mean% TrB cells of 1.982% ± 0.8242% vs. 1.434% ± 0.5672%). No differences were found in absolute TrB cell numbers (transitional B cells/µL of total blood) between groups (data not shown). TrB cell absolute numbers were not used for further analysis. We did not find significant differences between SKF and noBPAR or BPAR in other cell subsets (**Supplementary Figures 1**, **2**).

### 3.2 Transitional B-cell percentages at 3 months predict improved graft function up to 12 months post-transplantation

To assess the prospective predictive value of TrB cells at 3 months, we separately analyzed patients that were biopsied before and after 3 months. Of all patients with AKF (*n* = 22), 43% were biopsied after the third month, and of all patients with BPAR (*n* = 6), 66% were diagnosed after the third month post-transplantation.

**Figure 3:**
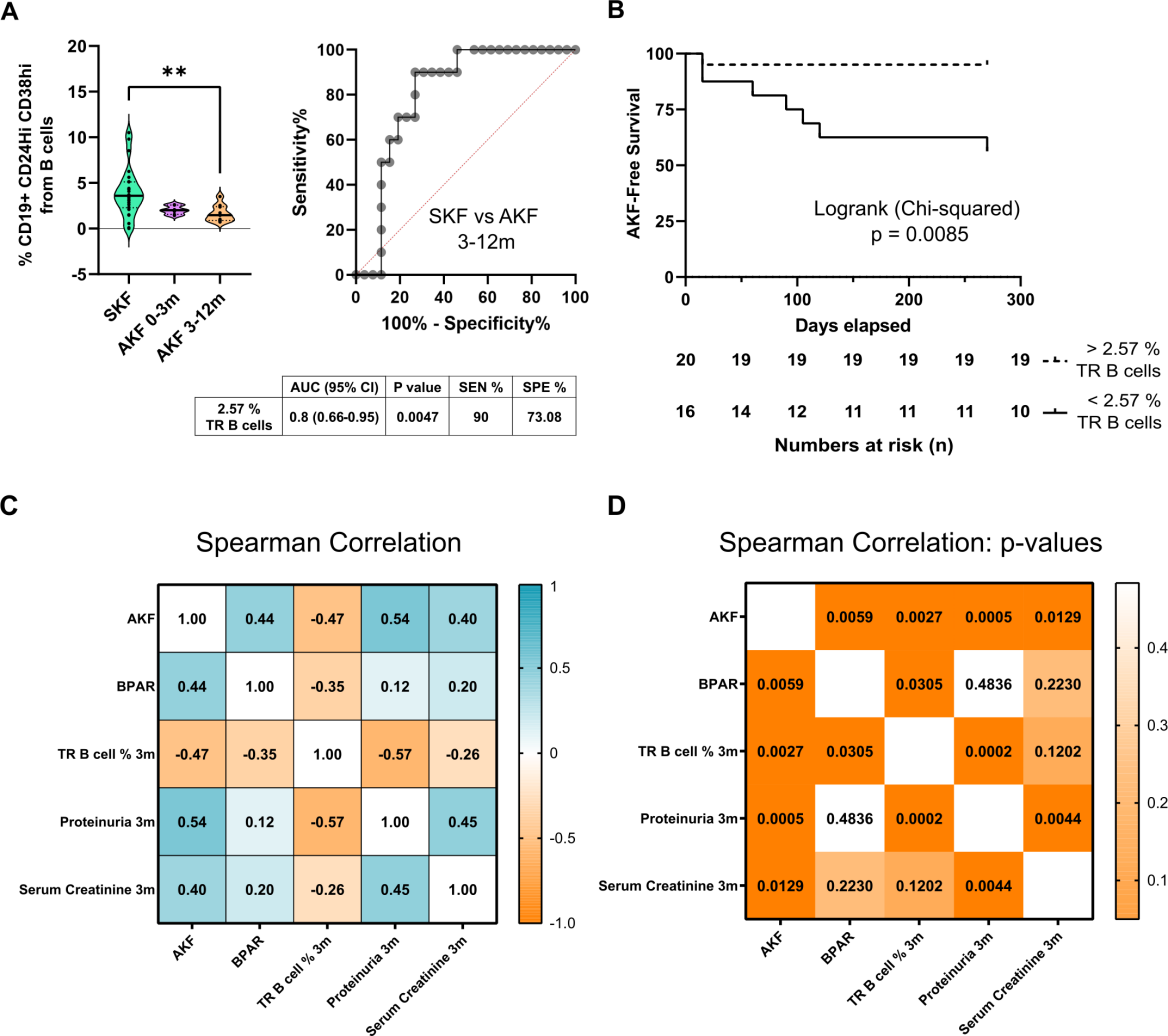
Transitional B-cell relative counts at 3 months predict worse graft outcome for the first-year follow-up. **(A)** Comparison of transitional B-cell (TrB cells) percentages stratifying patients with AKF according to diagnosis of altered kidney function during the first 3 months after transplantation (AKF 0–3m) or after 3 months (AKF 3–12m). TrB shows significantly higher percentages in patients with stable kidney graft function (SKF) compared to patients with altered kidney function (AKF) after 3 months with high AUC values, sensitivity, and specificity by ROC curve analysis. The table under graphs shows relevant statistical results from ROC curve analysis. **(B)** Survival curves show how patients with lower Breg percentages (<2.57%) have a significant decrease of AKF-free survival. Cutoff value (2.57) was established according to ROC curve analysis from 4A. **(C)** Spearman correlation *r* values of AKF, BPAR, transitional B cells (TrB cells), proteinuria, and creatinine at 3 months and **(D)** Spearman correlation *p*-values show how all parameters tested correlate significantly to AKF, but only TrB cells correlate to BPAR. **p < 0.01.

The % TrB cells at 3 months significantly discriminated patients with SKF from patients with AKF with an identified clinical event after the 3-month TrB analysis (*p* = 0.0056, Mean% TrB cells of 3.92% ± 2.6% vs. 1.65% ± 0.9%) (**Figure 3A**). ROC curve analysis showed a very sensitive and specific correlation between % TrB cells at 3 months and risk of AKF (**Figure 3A**) (AUC= 0.8, sensitivity = 90%, specificity = 73.08%, cutoff = 2.57%). Furthermore, survival curves for AKF-free survival stratifying patients between those with higher or lower TrB cells at 3 months (2.57%, value taken from the ROC curve analysis according to highest sensitivity and specificity values) significantly discriminated patients (log-rank *p* = 0.0165), with 90% of patients with higher TrB cell percentages showing stable function (**Figure 3B**).

To compare the potential of TrB cells to other clinically useful biomarkers, we performed correlation analysis for patients with AKF after the 3-month time point. All parameters at 3 months, TrB cells, serum creatinine, and proteinuria, were significantly correlated to patients with AKF during follow-up; however, only the % TrB cells at 3 months were significantly associated with BPAR (correlation: −0.35, *p* = 0.0305) (**Figures 3C, D**).

Finally, to discard potential confounders, we assessed the influence of the immunosuppressive regime and infectious events on lymphocyte subsets. Patients received one of the following induction therapies: rATG, basiliximab, or no induction therapy. rATG, as expected, had an influence in several CD3^+^ T-cell subsets maintaining CD4^+^ and CD8^+^ T cells low at all time points, while regarding the B-cell compartment, no differences were found between different induction therapies received at any time point (**Supplementary Figure 4**).

Most patients received maintenance therapy with PDN, FK, and MMF, while the rest received PDK, FK, and mTOR inhibitors. No differences were found in the absolute counts of any lymphocyte subsets relative to the maintenance therapy received at any time point (**Supplementary Figure 5**).

### 3.3 TrB analysis in frozen PBMCs is highly correlated to fresh samples and maintains prognostic values in 1-year allograft functional outcomes

**Figure 4:**
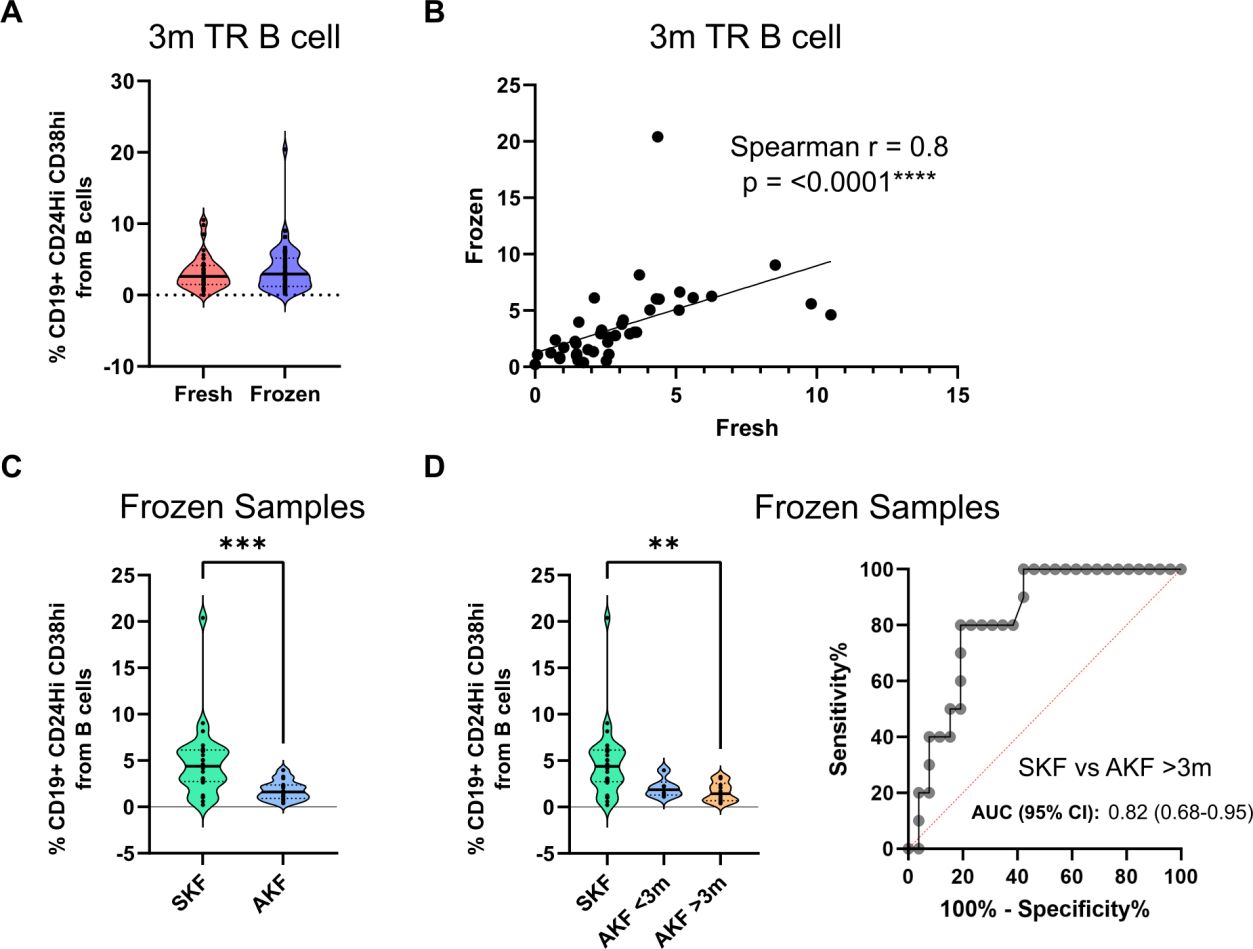
Transitional B-cell percentages are comparable in fresh and frozen samples and maintain their discriminative potential in frozen samples. **(A)** Comparison of transitional B-cell (TrB cell) percentages in fresh and frozen samples analysis showing no significant differences **(B)** Spearman correlation analysis of TrB cells of fresh and frozen analysis showing a significant and highly correlated result. **(C)** TrB cell percentage comparison between patients with stable kidney function (SKF) and altered kidney function (AKF) in frozen samples shows significantly elevated percentages in the SKF group. **(D)** Comparison of TrB cell percentages between patients according to the time of AFK diagnosis shows a trend of lower TrB percentages in patients with diagnosis of AKF before the 3-month time point (AKF <3m) and a significant decrease in patients with diagnosis afterwards (AKF >3m). **p < 0.01, ***p < 0.001.

Since the direct phenotyping was performed in fresh blood, we decided to evaluate the stability of the TrB immunophenotyping on the frozen PBMCs stored. We observed that the % TrB cells were stable and similar comparing fresh and frozen sample means (**Figure 4A**) with a correlation value of 0.8 (**Figure 4B**). As fresh and frozen samples were comparable, we tested whether we could use the frozen samples as a prognostic value to stratify 1-year patients with AKF and SKF. In frozen PBMCs, % TrB cells at 3 months showed comparable efficiency in stratifying patients with SKF and AKF (**Figure 4C**) and predicting AKF after 3 months (**Figure 4D**) (AUC = 0.82, sensitivity = 80%, specificity = 80.67%, cutoff = 2.505%). These comparable results between fresh and frozen samples validate the use of frozen samples from the patients in further analyses.

### 3.4 Novel TrB cell-associated markers are overexpressed in patients with higher TrB cells at 3 months

In a previous study, we conducted an in-depth characterization of *in vitro*-induced TrB cells, leading to the identification of a “TrB-associated gene signature” (20) composed of six human genes (ANXA1, CXCR4, CD229, FN1, TGFBI, and THBS1).

**Figure 5:**
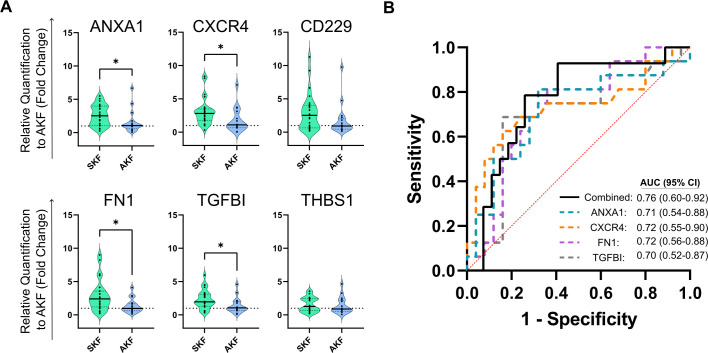
Transitional B cell-associated genes show a correlation at 3 months post-transplantation with altered kidney function **(A)** Violin plots show the relative expression to the altered kidney function (AKF) group mean value by the 2^–ΔΔCT^ method of single genes in PBMCs. Four out of six genes show a significant decrease in patients with AKF compared to patients with stable function during the first-year post-transplantation (SKF). **(B)** ROC curve analysis of single genes (dotted colored lines) or the combination of them by multiple logistic regression (black line) show high discrimination potential of TrB cell-associated genes. *p < 0.05.

To expand upon the results of the current study and to further evaluate the predictive value of circulating TrB cells in kidney transplant patients, we examined the “TrB-associated gene signature” in patients’ frozen PBMCs using quantitative PCR (qPCR). Among the six genes, four exhibited differential expression between patients with SKF and AKF (**Figure 5A**). Specifically, ANXA1, CXCR4, FN1, and TGFBI showed higher expression in patients with SKF (*p* = 0.0195, 0.0135, 0.0168, and 0.0286, respectively) while non-significant differences were observed in CD229 and THSB1 expression (*p* = 0.1 and 0.48, respectively). ROC curve analysis demonstrated a robust discrimination potential between the combined expression of these four genes at 3 months post-transplantation and the risk of impaired kidney function (**Figure 5B**), with an AUC of 0.76.

Furthermore, when patients with AKF were stratified into subgroups based on BPAR and noBPAR (**Supplementary Figure 6**), we found that CXCR4 expression was significantly elevated in patients with SKF compared to those with BPAR (*p* = 0.0022, 2.976 ± 1.961 vs. 0.54 ± 0.4679) while exhibiting comparable levels to the noBPAR group (*p* = 0.87, 2.976 ± 1.961 vs. 2.363 ± 1.94). No significant differences were observed in the expression of other markers.

On the other hand, we also tested IL-10 mRNA expression in our cohort. We did not find differences in the expression of IL-10 between SKF and AKF groups (*p* = 0.4668, 1.714 ± 1.348 vs. 1.402 ± 1.315, not shown on graph) or SKF and noBPAR or BPAR groups (*p* = 0.99 and 0.99, 1.617 ± 1.346 in SKF vs. 1.554 ± 1.564 and 1.068 ± 0.402 in noBPAR and BPAR, respectively, not shown on the graph).

### 3.5 TrB cell monitoring and molecular screening of TrB cells at 3 months predict long-term (>1 year) AKF

To assess the long-term predictive capacity of the 3-month post-transplantation TrB cell monitoring and gene signature beyond the primary time point of 12 months, we followed up the patients up to 5 years since the beginning of the study. After 12 months of follow-up, nine new patients underwent biopsy procedures due to clinical indications, and two of them received a diagnosis of acute rejection.

**Figure 6:**
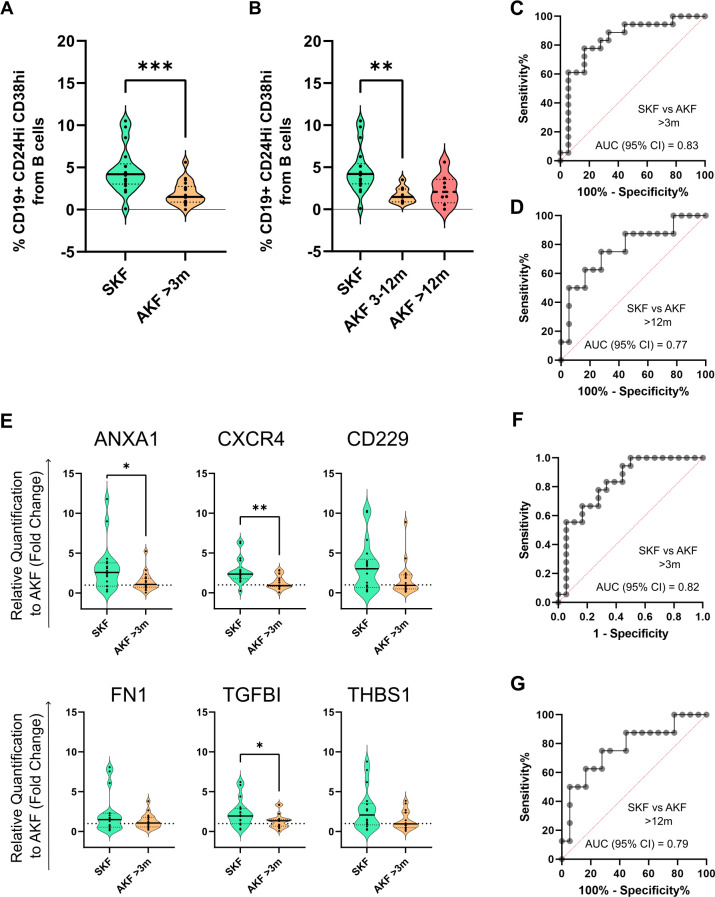
Transitional B-cell immunophenotyping and molecular analysis at 3 months post-transplantation maintain their discrimination potential beyond the 12-month time point **(A)** Violin plot shows the comparison of TrB cells between patients with stable kidney function (SKF) and patients with a diagnosis of altered kidney function (AKF) after the 3-month time point that were followed up until 5 years post-transplantation. **(B)** TrB cell percentage comparison between patients with AKF with diagnosis between 3 and 12 months (AKF 3–12m) and after the 12-month time point (AKF >12m). Results show a significant decrease in AKF 3–12m compared to SKF. **(C)** ROC curve analysis of TrB percentages between patients with SKF and AKF with diagnosis after 3 months. **(D)** ROC curve analysis of TrB cell percentages between patients with SKF and AKF with diagnosis after 12 months. **(E)** Violin plot shows the comparison of TrB cell-associated genes relative expression to the AKF group mean value by the 2^–ΔΔCT^ method between patients with SKF and patients with a diagnosis of AKF after the 3-month time point that were followed up until 5 years post-transplantation. **(F)** ROC curve analysis of the combination of ANXA1, CXCR4, FN1 and TGFBI by multiple logistic regression between patients with SKF and AKF with diagnosis after 3 months. **(G)** ROC curve analysis on the comparison of the same genes in patients with SKF and AKF with diagnosis after 12 months. *p < 0.05, **p < 0.01, ***p < 0.001.

Patients exhibiting AKF at 3 months post-transplantation displayed significantly lower TrB percentages compared to those with stable kidney function (*p* = 0.0003, Mean% TrB cells: 4.638% ± 2.676% vs. 1.954% ± 1.385%) (**Figure 6A**). Upon further stratification of AKF cases between 3 and 12 months post-transplantation and those manifesting AKF beyond 12 months (**Figure 6B**), a markedly diminished expression was observed in the former group compared to SKF (*p* = 0.0021, Mean% TrB cells of 4.638% ± 2.676% vs. 1.655% ± 0.901%). Patients with AKF beyond 12 months exhibited a non-significant trend towards lower TrB % compared to SKF (*p* = 0.08, Mean% TrB cells of 4.638% ± 2.676% vs. 2.326% ± 1.825%). ROC curve analysis showed high predictive values for TrB cells for AKF after 3 months (**Figure 6C**) and 12 months (**Figure 6D**) post-transplantation, yielding AUC values of 0.83 and 0.77, respectively.

Regarding the TrB-associated gene markers, ANXA1, CXCR4, and TGFBI exhibited significant differences (*p* = 0.0187, 0.0017, and 0.04, respectively), and their higher expression in circulation was associated to patients with stable kidney function up to 5 years of follow-up (**Figure 6E**). Similarly, ROC curve analysis showed high predictive values of TrB cells for AKF after 3 months (**Figure 6F**) and after 12 months (**Figure 6G**) with AUC values of 0.82 and 0.79, respectively.

## 4 Discussion

Regulatory B cells (Bregs) have been a topic of interest in kidney transplantation due to their association to graft tolerance (12, 13). However, elucidating their precise role in human disease and establishing robust methodologies for their identification and application remain a challenge. In this work, we hypothesized that the characterization of Breg subsets via direct immunophenotyping (transitional B cells: CD19^+^CD24^hi^CD38^hi^) and the study of transitional B cell (TrB)-associated molecular markers may be a promising tool to stratify kidney transplant patients according to their risk of rejection or allograft outcome.

Our results positively associated a transitional B cell (TrB cell)-combined signature at 3 months post-transplantation to favorable graft outcomes, coupled with reduced risk of rejection, underscoring the potential of TrB cells as a valuable prognostic biomarker in kidney transplant.

Patients that exhibited AKF during follow-up had a lower percentage of TrB cells at 3 months compared to patients who had stable graft function (SKF) throughout the first year post-transplantation. Notably, this statistically significant difference persisted after excluding patients with AKF with events before 3 months from the analysis, highlighting TrB cells’ prospective predictive capabilities. At 3 months, a TrB cell percentage of 2.57% from total B cells accurately predicted graft dysfunction within the first year. However, we did not observe statistically significant differences in TrB cells at 3 months between non-rejection patients with graft dysfunction (noBPAR) to patients with BPAR, which might be attributed to the limited number of patients with BPAR.

These findings suggest that TrB cells may play an important role in regulating the immune response following transplantation. It has been described that B-cell repopulation returns towards baseline by month 3 (17), indicating proper immune homeostasis in kidney transplanted patients. This time frame aligns with the time point in our study, highlighting the pivotal role of the first few months in the restoration of immune function. Interestingly, we observed that the discriminatory power of TrB cells extended beyond the initial year post-transplantation, persisting for up to 5 years of follow-up.

Our results are in line with the results published by Shabir et al. (16) who showed that 50% of patients with pre-transplant TrB cells (CD19^+^CD24^hi^CD38^hi^) <1% had rejection while no patients with a frequency of >3% had rejection. In the same line, Cherukuri et al. (23) were able to demonstrate that patients with rejection had the lowest levels of Bregs (high IL-10/TNF-α ratio in CD19^+^CD24^hi^CD38^hi^) compared to patients with graft dysfunction but no rejection, stable graft function, and healthy volunteers, comparable to what Luo et al. (24) found related to antibody-mediated rejection (AMR). In this study, AMR incidence was correlated to higher *in vitro*-activated IL10+ Bregs at 90 days post-transplantation. Svachova et al. (18) also documented a decline in absolute numbers of TrB cells at month 3 and found a correlation between lower TrB cells and higher rejection risk, similar to the trend observed in our study.

However, it is important to mention that different time points have been described to be relevant for TrB cells in kidney transplantation. Shabir et al. (16) showed that non-rejecting patients had increased TrB cells at pre-transplant and 14 days post-transplantation compared to rejectors. On the other hand, Laguna-Goya et al. (25) demonstrated that higher frequencies of *in vitro* stimulated IL-10+ Bregs cells before transplantation, compared to day 7 post-transplantation, were an independent risk factor for acute rejection and graft failure. These differences might be explained by different cohort characteristics and different methods of isolating and analyzing TrB/Breg cells.

Despite differences on time points and Bregs characterization method, the common bottom line of our study and the ones already mentioned is that TrB cells decrease after transplantation favors alloreactivity, highlighting their influence on graft outcome, and giving consistency to an emerging field of interest in kidney transplantation. Multicenter studies remain a need in the field to validate the application of the B-cell subset phenotype in solid organ transplantation, given the similar and consistent observations made in different studies as previously discussed. As this initial reduction of TrB cells might be related to immunosuppression treatment (5), we analyzed the impact that immunosuppressive therapeutic agents used in our study might have on TrB cells. We did not find any difference in absolute counts and TrB cell percentage at any time point between different induction or maintenance therapy received. These results are in line with other studies in the field (18, 19, 26). However, this may be due to the homogeneous immunosuppressive drug regimens used in these studies. Immunosuppressive drugs and other immunotherapies have been reported to modulate Breg numbers (5). Monitoring Breg levels could stratify patients according to their graft outcome risk and potentially pave the way for therapeutic interventions aimed at boosting this B-cell subpopulation to promote immune homeostasis and graft function.

Regarding the phenotypic markers used to define Bregs in our study, we aimed to analyze the TrB cell phenotype based on the CD19^+^CD24^hi^CD38^hi^ signature. Even though we are aware of the recent interest on measuring cytokine profiles of Bregs (IL-10 and TNF-α) (23), this analysis was beyond the scope of this study. This cytokine ratio is in line with the results that our group has recently published, pointing at the dual role of IL-10 secretion by TrB cells (20). To study IL-10 secretion, *in vitro* stimulation is required, making the study of pro-inflammatory cytokines like TNF-α mandatory to fully understand its immunoregulatory potential. However, in this study, we initially propose direct immunophenotyping as a more efficient and clinically applicable alternative to the proposed cytokine balance study, which typically involves time-consuming and expensive lymphocyte culture and *in vitro* activation. Direct whole blood phenotyping, once standardized, offers a high-throughput methodology for analyzing patient data aligned with clinical needs. Moreover, we also assessed and validated the immunophenotyping of frozen PBMCs for all patients. We observed that the proportion of the different subsets was comparable, and in particular, the TrB cell percentage correlated to the values assessed in fresh blood samples. This is a key finding in order to compare studies using fresh or frozen samples and also for further validation of the TrB phenotype in frozen samples from different cohorts.

Besides the immunophenotyping, we evaluated TrB-associated markers by gene expression analysis in PBMCs. We have previously described a set of genes (20) differentially expressed in induced Bregs (iBregs). We observed that the iBregs showing inhibitory potential were those that did not express high levels of IL-10+ upon activation and that blockage of TGF-β signaling inhibited regulatory B-cell induction and function, thus constituting a potential biomarker for kidney transplantation monitoring. Those cells overexpressed several genes and we selected a set of six representatives to validate in the current cohort. In this study, we adopted a bottom-up approach, starting with the most straightforward and translational method of using PBMCs directly to limit the need for the costly isolation of B cells from patient samples. Interestingly, we were able to find differential expression at 3 months post-transplantation of four genes, ANXA1, CXCR4, FN1, and TGFBI, between patients with SKF and patients with AKF during the first year of transplantation, which is in line with the results of the TrB cells’ analysis. This finding serves as a proof of concept for the potential of the newly identified iBreg-associated molecular signature and complements the hypothesis that B cells or specific B-cell signatures could be potential markers of graft function. This strategy follows the same scope as the direct immunophenotyping of TrB cells and the comparison of fresh and frozen samples, with a focus on the translation potential of the B-cell study for application in a clinical setting. In addition, PBMCs are routinely isolated and stored in biobanks and could be employed in additional studies without the need for additional processing in retrospective studies.

From this gene set, FN1 (Fibronectin 1) and TGFBI (Transforming Growth Factor Beta-Induced) are major constituents of extracellular matrices and accumulate within kidney tissue throughout the fibrotic process development. Fibronectin has multiregulatory functions, being involved in coagulation and the regulation of transforming growth factor beta (TGF-β), among other cell signaling pathways through the binding of integrins (27). TGFBI, on the other hand, is an extracellular matrix protein induced by TGF-β, playing a crucial role in cancer progression, including cell proliferation, angiogenesis, and apoptosis (28). TGFBI also exhibits immunosuppressive effects within its microenvironment. Both FN1 and TGFBI are produced by macrophages (29, 30), and their production is associated with the development of immunosuppressive microenvironments and antifibrotic effects (31, 32). Low expression of FN1 is found in T cells from healthy donors, and it is upregulated upon activation (33). However, the role of FN1 and TGFBI in other PBMC subtypes, such as B cells, or their involvement in kidney transplant patients, remains to be explored.

While the specific role that FN1 and TGFBI play in the regulatory properties of TrB cells or their relation to graft function in high-TrB patients remains to be elucidated, these proteins are involved downstream in the TGF-β signaling pathway. *In vitro* blockade of TGF-β signaling (20) showed that the induction and function of regulatory B cells depend on TGF-β signaling, highlighting the importance of this pathway. In contrast, IL-10 blockage showed no alteration in the function of regulatory B cells, aligning with recent studies that explore the potential of IL-10-independent B cells as biomarkers of kidney graft functions, and instead highlighting the importance of granzyme-B+ plasma cells (4, 34). In this line, we also analyzed IL-10 expression by qPCR in patients’ PBMCs and we did not find differences, supporting the need for intracellular staining to discriminate patients according to its expression, as previously published by other groups (23) and already discussed in this manuscript.

Additionally, Annexin A1 (ANXA1) is a protein suggested as a regulator of inflammation (35), with significant functions in the kidney, mainly by inhibiting cytosolic phospholipase A2 (PLA2) and reducing leucocyte migration in response to cytokines and chemokines in animal models of inflammation. CXCR4 expression in allograft tissue has been extensively associated with chronic allograft alterations. The CXCL12/CXCR4 pathway can promote chronic allogeneic nephropathy progression and fibrosis by recruiting bone marrow-derived cells to the kidney. Several studies have targeted CXCR4 as a mediator of T-cell recruitment in kidney tissue. Conversely, other studies have linked CXCR4 to pathways related to cell survival (36), migration, angiogenesis, renoprotection (37), and Treg function in renal cancer (38). Additionally, SDF-1 (the CXCR4 ligand) expression in ischemic kidney has been associated with the recruitment of CXCR4-expressing cells to the kidney (39) and may constitute a signal involved in kidney wound healing and homeostasis.

Overall, these proteins point to novel physiological pathways involved in inflammation that could mediate graft function. Future studies will be needed to elucidate the direct effect of these markers on the function of regulatory B cells, their expression on TrB cells or other PBMC subsets, and their relation to TrB cells and graft function *in vivo*. This will be a needed step in validating the results of the study that fall outside the scope of this manuscript.

One limitation of the current study was the lack of biopsies in the group with stable kidney function. Our center does not perform protocol biopsies after kidney transplantation, which could hinder our ability to detect patients with subclinical rejection that were not identified as AKF. In this line, we can observe how, in long-term follow-up (up to 5 years), the discriminatory potential of TrB cells is maintained with a trend of lower levels of TrB cells in patients with graft dysfunction beyond the first year post-transplantation. This may represent patients with SKF with subclinical rejection during follow-up that have a detectable alteration in the TrB compartment at 3 months and underscores the early predictive potential of TrB analysis. Further validations must be performed to confirm this hypothesis. In addition, while IL-10 was not differentially expressed in patients’ PBMCs, we did not investigate the role of cytokines in other compartments. Future studies should investigate if IL-10 or other immunoregulatory cytokines, such as TGF-β, are upregulated in serum or graft tissue, and could be correlated or mediate improved graft function in high-TrB and/or iBreg signature expressing patients.

In summary, the measurement of CD19^+^CD24^hi^CD38^hi^ TrB cells at 3 months by direct immunophenotyping and by studying molecular markers stratifies patients according to risk of graft outcome and rejection during the first year after transplantation, allowing, on the one hand, the identification of low-risk patients who can benefit from the minimization of immunosuppression regimens and, on the other hand, the identification of high-risk patients who might benefit from a closer follow-up and preemptive strategies to avoid graft dysfunction and rejection.

## Data Availability

The raw data supporting the conclusions of this article will be made available by the authors, without undue reservation.

## Supplementary material

Supplementary Figure 1 T-cell populations do not discriminate graft function. Violin plots show the absolute numbers of different T-cell absolute cell counts. Total T cells, CD4+ T cells, and CD8+ T cells are reduced at 1-week post-transplantation, and steadily recover pre-transplantation number by month 12 post-transplantation. No differences between SKF, noBPAR, and BPAR groups are observed at any time point. **p* < 0.05, ***p* < 0.01, ****p* < 0.001, *****p* < 0.0001.

Supplementary Figure 2 Naïve (CD19+CD27-IgD+), memory (CD19+CD27+IgD-), and total B cells (CD19+) do not discriminate graft function. Violin plots show the absolute numbers of different B-cell absolute cell counts. No differences between SKF, noBPAR, and BPAR groups are observed at any time point for the represented populations. Memory B cells are increased at 1-week post-transplantation. **p* < 0.05, ***p* < 0.01, ****p* < 0.001, *****p* < 0.0001.

Supplementary Figure 3 Transitional B-cell absolute cell counts at 3 months show less discriminatory potential of graft outcome than TrB cell percentages. **(A)** Comparison of transitional B-cell (TrB cells) absolute cell numbers stratifying patients with AKF according to diagnosis of altered kidney function during the first 3 months after transplantation (AKF 0–3m) or after 3 months (AKF 3–12m). TrB shows non-significant higher numbers in patients with stable kidney graft function (SKF) compared to patients with altered kidney function (AKF) after 3 months with moderate AUC values, sensitivity, and specificity by ROC curve analysis. The table under graphs shows relevant statistical results from ROC curve analysis.

Supplementary Figure 4 Thymoglobulin induces severe total T-cell and CD4+ T-cell depletion that is restored after 6 months. Violin plots show the absolute numbers of different T-cell and B-cell subpopulations during the first year after transplantation when stratifying patients according to the induction immunosuppressive treatment. **p* < 0.05, ***p* < 0.01, ****p* < 0.001, *****p* < 0.0001.

Supplementary Figure 5 Absolute cell numbers show no differences when comparing different immunosuppressive drug regimes. Violin plots show the absolute numbers of different T-cell and B-cell subpopulations during the first year after transplantation when stratifying patients according to the maintenance immunosuppressive treatment. **p* < 0.05, ***p* < 0.01, ****p* < 0.001, *****p* < 0.0001.

Supplementary Figure 6 Transitional B cell-associated genes show a trend of lower expression at 3 months post-transplantation in patients with altered kidney function, and CXCR4 is significantly reduced in patients with biopsy-proven acute rejection **(A)** Violin plots show the relative expression by the 2^—ΔΔCT^ method of single genes in PBMCs. CXCR4 is significantly decreased in patients with biopsy-proven acute rejection (BPAR) compared to patients with stable function during the first-year post-transplantation (SKF). ***p* < 0.01.

Supplementary Table 1 List of labeling antibodies used for fresh whole blood flow cytometry. Columns indicate antibodies used for the labeling of samples in each tube processed. One microliter of each antibody was used per tube.

Supplementary Table 2 List of qPCR primer pairs used. The first column indicates the full name and gene symbol of the sequence that each primer pair is specific for. The second column indicates the primer pair sequences.

Supplementary Table 3 Renal biopsy diagnosis. Chronic transplant glomerulopathy and secondary focal segmental glomerulosclerosis. Values expressed as total number and (%).
