# Supplementary figures and images for: Molecular screening of transitional B cells as a prognostic marker of improved graft outcome and reduced rejection risk in kidney transplant
Source: Front Immunol. 2024 Aug 12;15:1433832. doi: 10.3389/fimmu.2024.1433832 (PMC11348389; doi:10.3389/fimmu.2024.1433832)

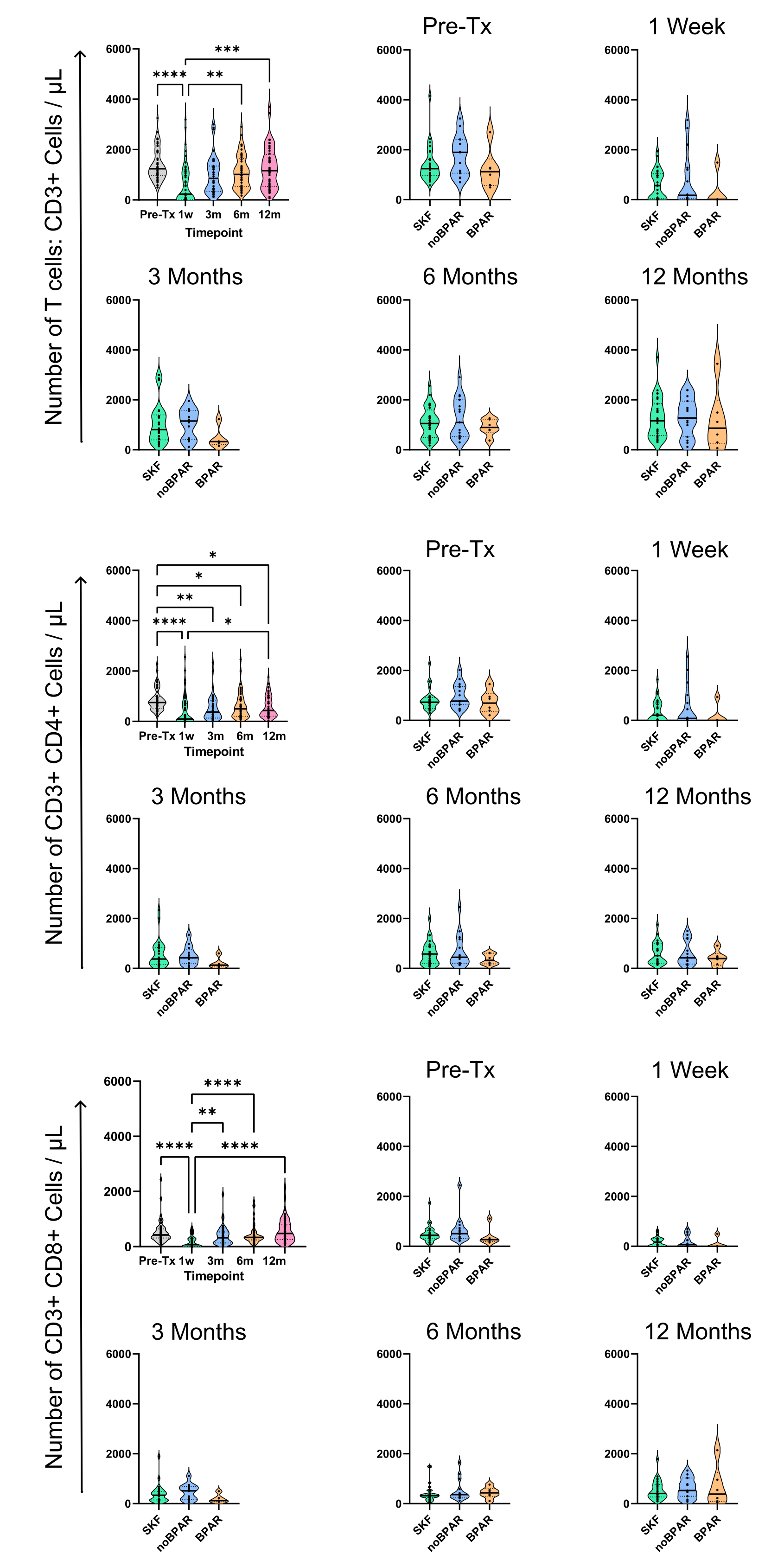

Supplement: Supplementary Figure 1 — T-cell populations do not discriminate graft function. Violin plots show the absolute numbers of different T-cell absolute cell counts. Total T cells, CD4+ T cells, and CD8+ T cells are reduced at 1-week post-transplantation, and steadily recover pre-transplantation number by month 12 post-transplantation. No differences between SKF, noBPAR, and BPAR groups are observed at any time point. *p < 0.05, **p < 0.01, ***p < 0.001, ****p < 0.0001. [file Image1.tiff]

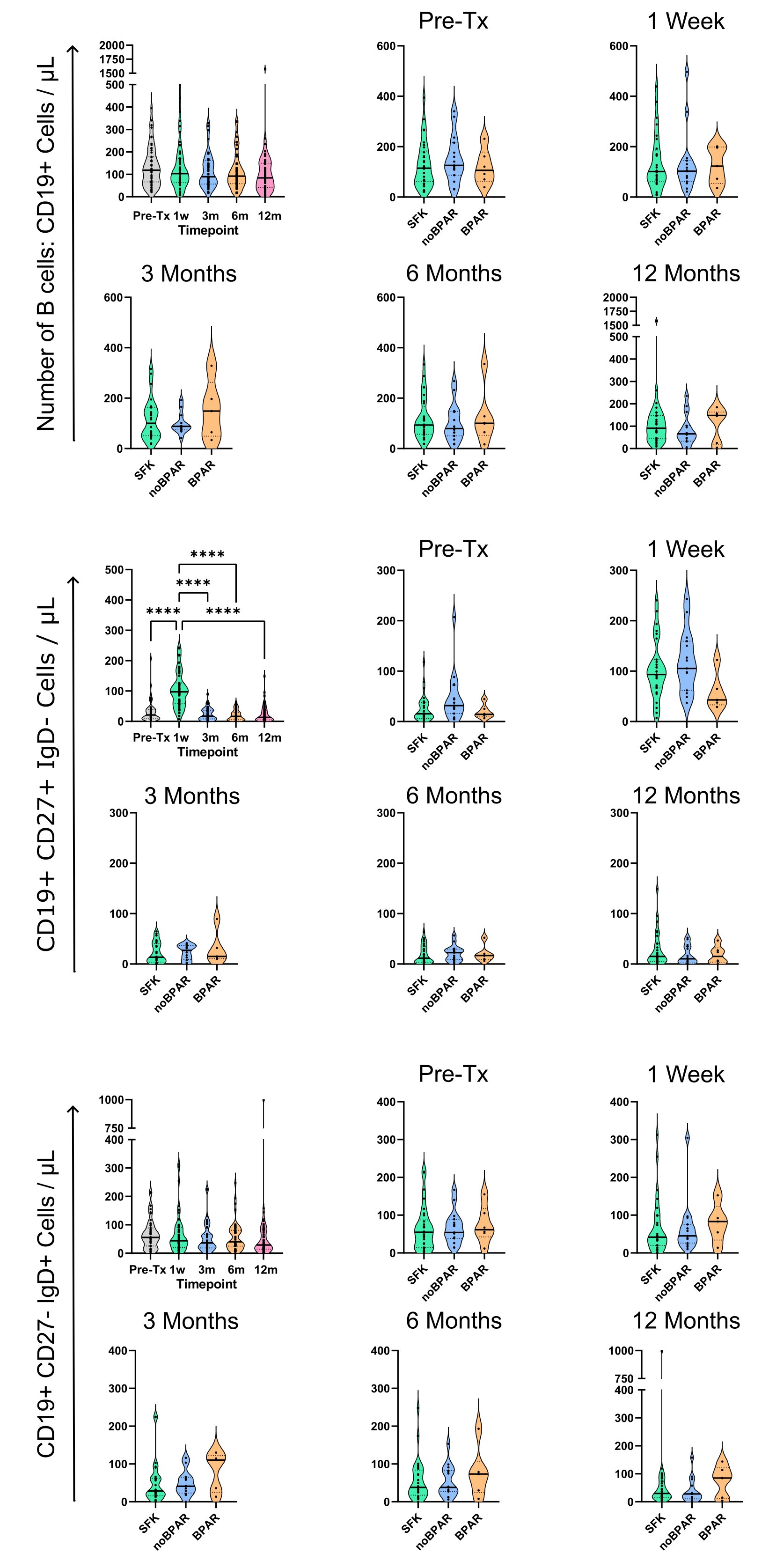

Supplement: Supplementary Figure 2 — Naïve (CD19+CD27-IgD+), memory (CD19+CD27+IgD-), and total B cells (CD19+) do not discriminate graft function. Violin plots show the absolute numbers of different B-cell absolute cell counts. No differences between SKF, noBPAR, and BPAR groups are observed at any time point for the represented populations. Memory B cells are increased at 1-week post-transplantation. *p < 0.05, **p < 0.01, ***p < 0.001, ****p < 0.0001. [file Image2.tiff]

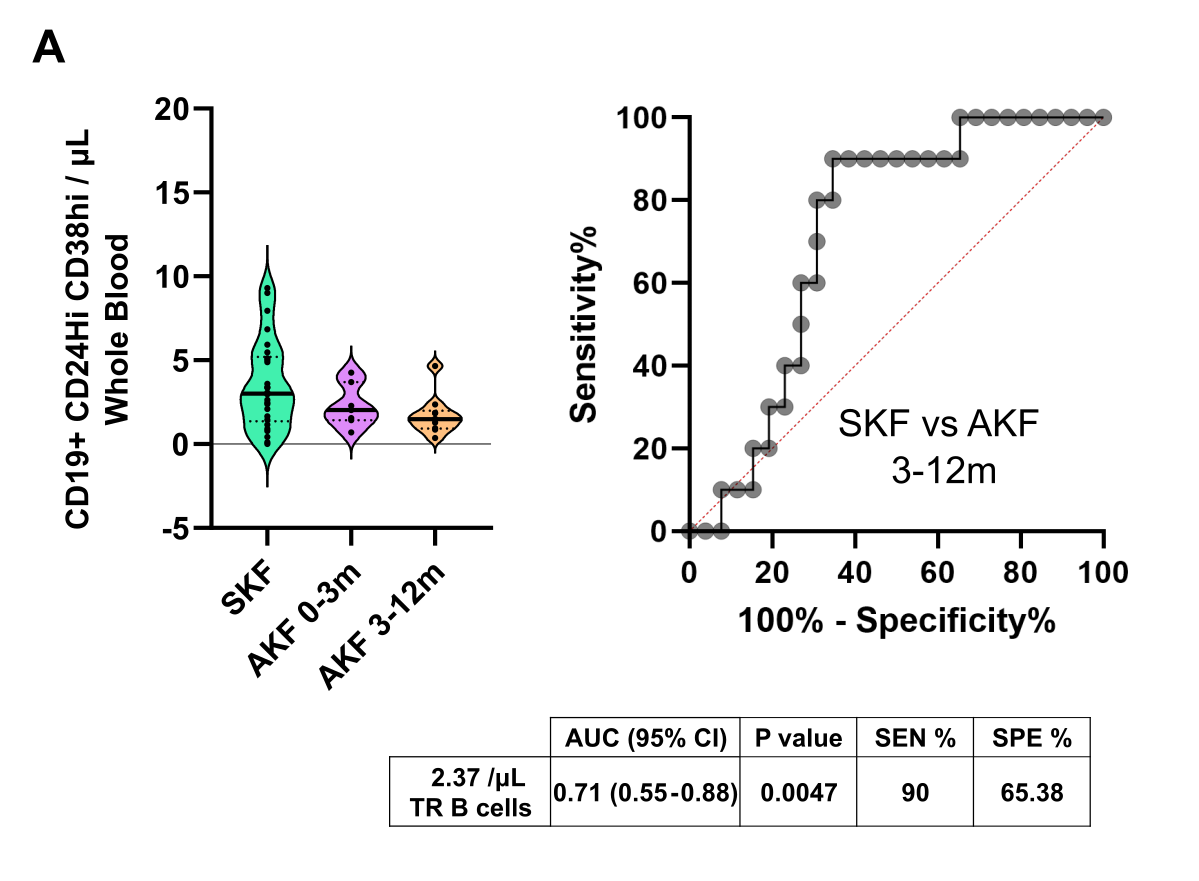

Supplement: Supplementary Figure 3 — Transitional B-cell absolute cell counts at 3 months show less discriminatory potential of graft outcome than TrB cell percentages. (A) Comparison of transitional B-cell (TrB cells) absolute cell numbers stratifying patients with AKF according to diagnosis of altered kidney function during the first 3 months after transplantation (AKF 0–3m) or after 3 months (AKF 3–12m). TrB shows non-significant higher numbers in patients with stable kidney graft function (SKF) compared to patients with altered kidney function (AKF) after 3 months with moderate AUC values, sensitivity, and specificity by ROC curve analysis. The table under graphs shows relevant statistical results from ROC curve analysis. [file Image3.tiff]

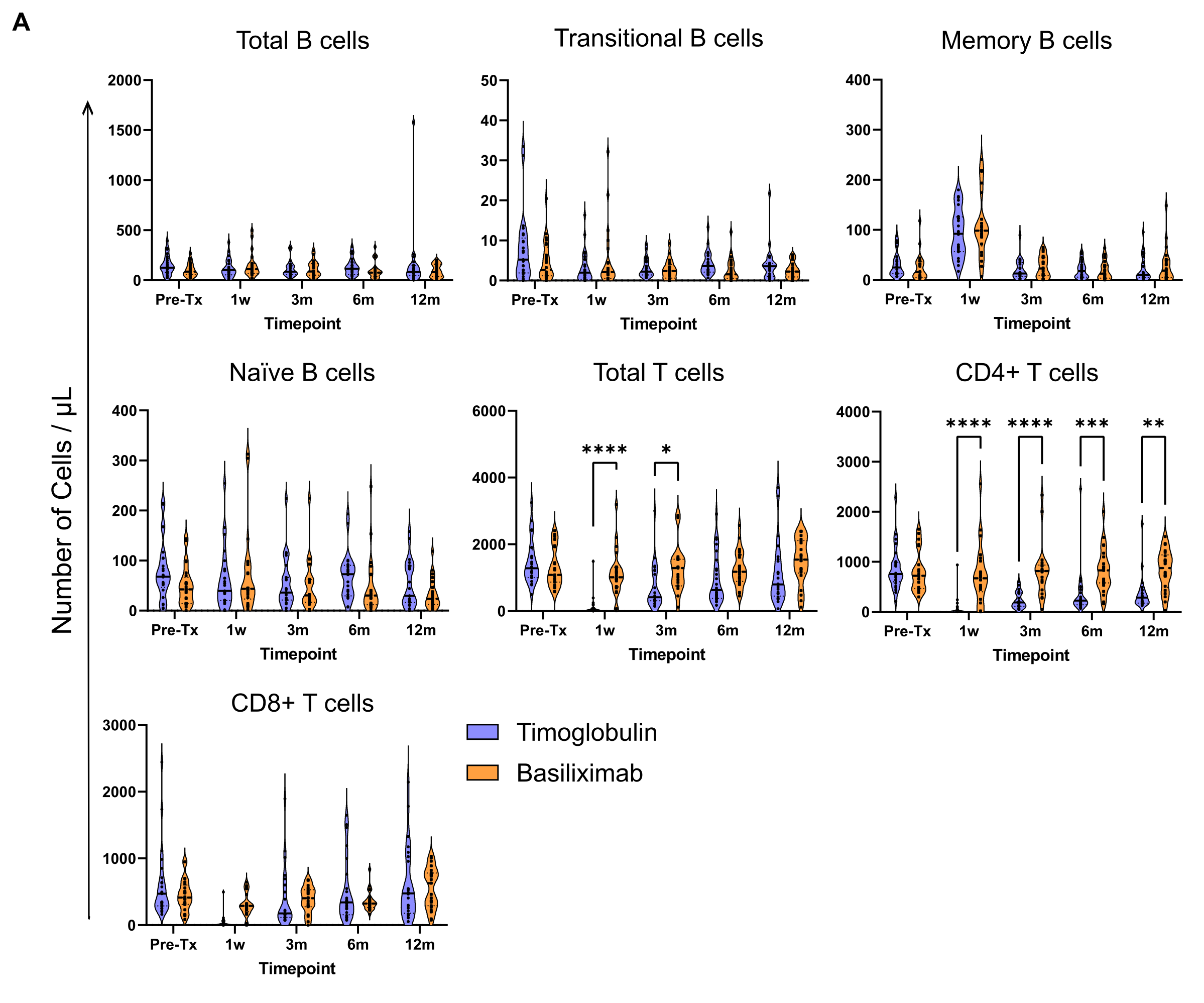

Supplement: Supplementary Figure 4 — Thymoglobulin induces severe total T-cell and CD4+ T-cell depletion that is restored after 6 months. Violin plots show the absolute numbers of different T-cell and B-cell subpopulations during the first year after transplantation when stratifying patients according to the induction immunosuppressive treatment. *p < 0.05, **p < 0.01, ***p < 0.001, ****p < 0.0001. [file Image4.tiff]

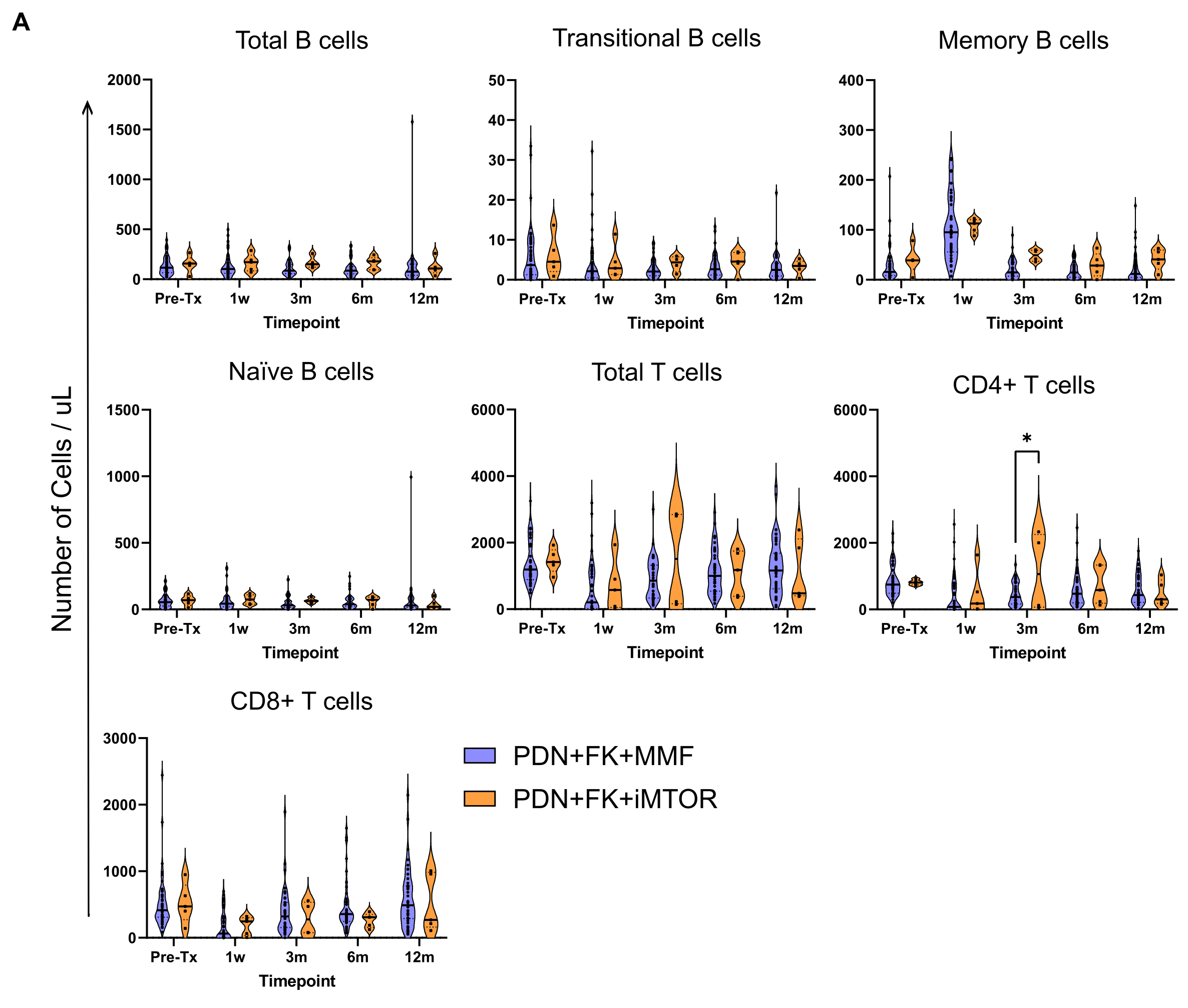

Supplement: Supplementary Figure 5 — Absolute cell numbers show no differences when comparing different immunosuppressive drug regimes. Violin plots show the absolute numbers of different T-cell and B-cell subpopulations during the first year after transplantation when stratifying patients according to the maintenance immunosuppressive treatment. *p < 0.05, **p < 0.01, ***p < 0.001, ****p < 0.0001. [file Image5.tiff]

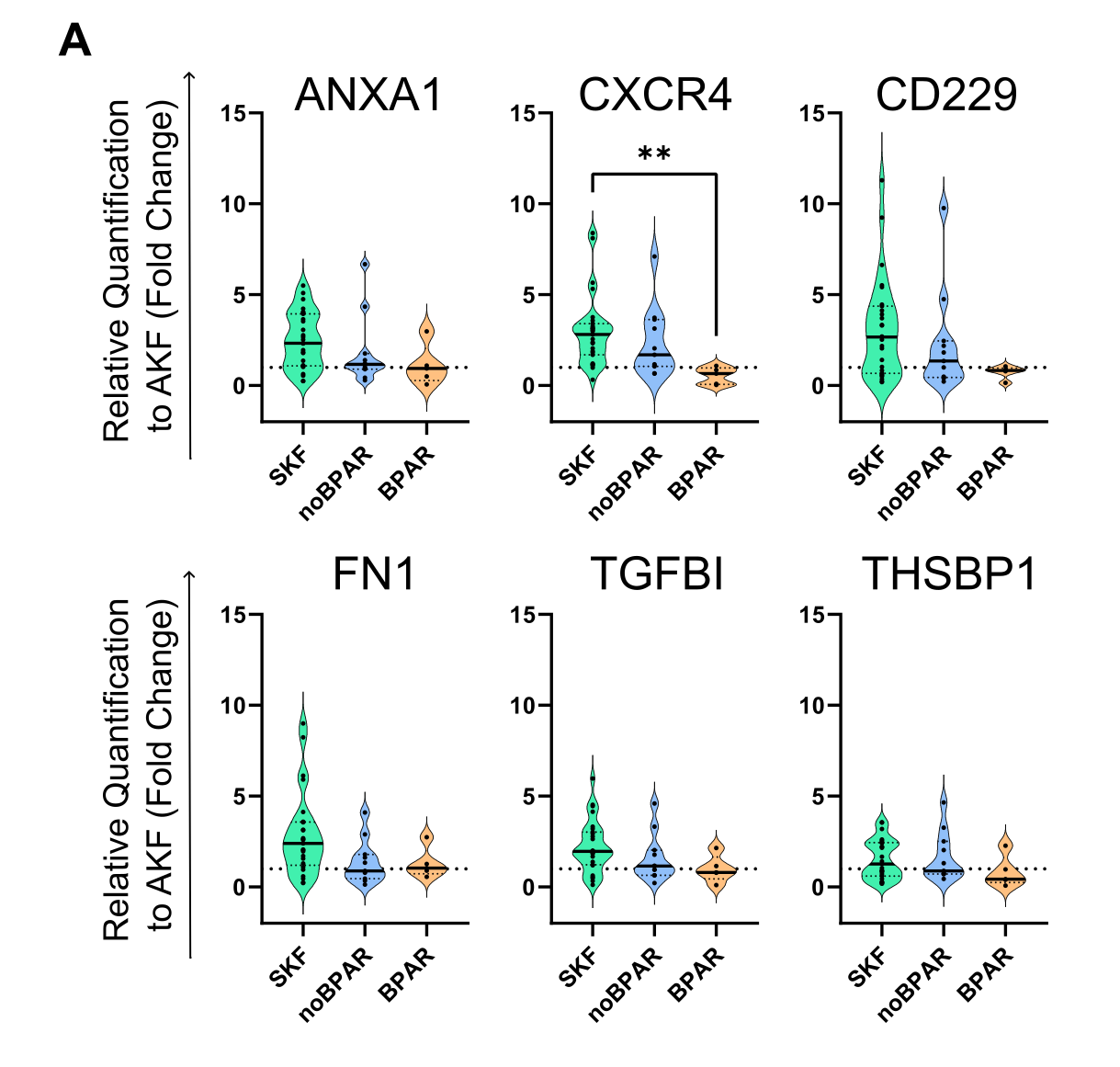

Supplement: Supplementary Figure 6 — Transitional B cell-associated genes show a trend of lower expression at 3 months post-transplantation in patients with altered kidney function, and CXCR4 is significantly reduced in patients with biopsy-proven acute rejection (A) Violin plots show the relative expression by the 2—ΔΔCT method of single genes in PBMCs. CXCR4 is significantly decreased in patients with biopsy-proven acute rejection (BPAR) compared to patients with stable function during the first-year post-transplantation (SKF). **p < 0.01. [file Image6.tiff]
