# Supplementary material for: Molecular screening of transitional B cells as a prognostic marker of improved graft outcome and reduced rejection risk in kidney transplant
Source: Front Immunol. 2024 Aug 12;15:1433832. doi: 10.3389/fimmu.2024.1433832 (PMC11348389; doi:10.3389/fimmu.2024.1433832)
Supplement: Supplementary Table 1 — List of labeling antibodies used for fresh whole blood flow cytometry. Columns indicate antibodies used for the labeling of samples in each tube processed. One microliter of each antibody was used per tube. [file Table1.docx]

**Supplementary Table 1. List of labelling antibodies used for fresh whole blood flow cytometry.** Columns indicate antibodies used for the labeling of samples in each tube processed. 1 uL of each antibody was used per tube.

| **Tube 1** | **Tube 2** |
| --- | --- |
| CD3-PerCp (BD Pharmingen, clone SP34-2) | CD3-PerCp (BD Pharmingen, clone SP34-2) |
| CD4-PE (BD Pharmingen, clone OKT4) | CD19-BV510 (BD Horizon, clone SJ25C1) |
| CD8-APC (BD Pharmingen, clone RPA-T8) | CD24-APC (Invitrogen, clone eBioSN3) |
| CD19-BV510 (BD Horizon, clone SJ25C1) | CD27-PE-Cy7 (Invitrogen, clone 0323) |
| CD45-FITC (BD Pharmingen, clone HI30) | CD38-PE (Invitrogen, clone HB7) |
| Perfect-Count Microspheres™ (Cytognos) | CD45-FITC (BD Pharmingen, clone HI30) |
|  | IgD-APC-Cy7 (BioLegend, clone IA6-2) |

**Supplementary Table 2. List of qPCR primer pairs used.** The first column indicates the full name and gene symbol of the sequence that each primer pair is specific for. The second column indicates the primer pair sequences.

| **Gene** | **Sequence (5’-3’)** | **Template Strand** |
| --- | --- | --- |
| Fibronectin (FN1) | AACCTTGCTCCTGACAGCTC | Forward |
|  | TTGGTGGGCTGACATTCTCC | Reverse |
| Thrombospondin 1  (THBS1) | GGACTCTGACGGCGATGGTC | Forward |
|  | ATCGGCGGAAATCGGTCTC | Reverse |
| Transforming growth factor beta induced  (TGFBI) | TGCTCCCACAAATGAAGCCT | Forward |
|  | GCCTCCGCTAACCAGGATTT | Reverse |
| Annexin A1  (ANXA1) | GTGAAGTGCGCCACAAGCAA | Forward |
|  | GGCGAGTTCCAACACCTTTCA | Reverse |
| Lymphocyte antigen 9  (LY9) | CTTCCTGCTCATGGGACTAAGA | Forward |
|  | TGGGACCAATCCAGATGACG | Reverse |
| C-X-C motif chemokine receptor 4  (CXCR4) | GAAACCCTCAGCGTCTCAGT | Forward |
|  | AGTAGTGGGCTAAGGGCACA | Reverse |
| Glyceraldehyde-3-phosphate dehydrogenase  (GAPDH) | GGCTTCAACTTAGACGCGGAG | Forward |
|  | TGGCTGGTATTAGCCTTGGGT | Reverse |

**Supplementary Table 3. Renal Biopsy Diagnosis.** Chronic transplant glomerulopathy and secondary focal segmental glomerulosclerosis. Values expressed as total number and (%).

| **KIDNEY BIOPSIES HISTOLOGICAL DIAGNOSIS** | **N TOTAL = 22** |
| --- | --- |
| ACUTE CELLULAR REJECTION  *IB*  *IIA* | 1 (2%)  4 (7.8%) |
| MIXED ACUTE REJECTION | 1 (2%) |
| ACUTE TUBULAR NECROSIS | 7 (31.8%) |
| RECURRENCE OF GLOMERULAR DISEASE | 1 (2%) |
| CALCINEURINE INHIBITORS TOXICITY | 2 (4%) |
| TROMBOTHIC MICROANGIOPATHY | 1 (2%) |
| OTHERS^+^ | 5 (9.8%) |
